# A scoping review of the use of quality improvement methods by community organizations in the United States, Australia, New Zealand, and Canada to improve health and well-being in community settings

**DOI:** 10.1093/ijcoms/lyab019

**Published:** 2022-04-21

**Authors:** Mallory Turner, Tara Carr, Randall John, Rohit Ramaswamy

**Affiliations:** Department of Maternal and Child Health, University of North Carolina at Chapel Hill Gillings School of Global Public Health, Chapel Hill, NC, USA; Department of Nutrition, University of North Carolina at Chapel Hill Gillings School of Global Public Health, Chapel Hill, NC, USA; Department of Health Policy and Management, University of North Carolina at Chapel Hill Gillings School of Global Public Health, Chapel Hill, NC, USA; Cincinnati Children’s Hospital Medical Center, Anderson Center for Health Systems Excellence, Cincinnati, OH, USA

**Keywords:** quality improvement, community health, capacity building, community capacity, health equity

## Abstract

**Background:**

Health-care facilities have used quality improvement (QI) methods extensively to improve quality of care. However, addressing complex public health issues such as coronavirus disease 2019 and their underlying structural determinants requires community-level innovations beyond health care. Building community organizations’ capacity to use QI methods is a promising approach to improving community health and well-being.

**Objectives:**

We explore how community health improvement has been defined in the literature, the extent to which community organizations have knowledge and skill in QI and how communities have used QI to drive community-level improvements.

**Methods:**

Per a published study protocol, we searched Scopus, Web of Science, and Proquest Health management for articles between 2000 and 2019 from USA, Australia, New Zealand, and Canada. We included articles describing any QI intervention in a community setting to improve community well-being. We screened, extracted, and synthesized data. We performed a quantitative tabulation and a thematic analysis to summarize results.

**Results:**

Thirty-two articles met inclusion criteria, with 31 set in the USA. QI approaches at the community level were the same as those used in clinical settings, and many involved multifaceted interventions targeting chronic disease management or health promotion, especially among minority and low-income communities. There was little discussion on how well these methods worked in community settings or whether they required adaptations for use by community organizations. Moreover, decision-making authority over project design and implementation was typically vested in organizations outside the community and did not contribute to strengthening the capability of community organizations to undertake QI independently.

**Conclusion:**

Most QI initiatives undertaken in communities are extensions of projects in health-care settings and are not led by community residents. There is urgent need for additional research on whether community organizations can use these methods independently to tackle complex public health problems that extend beyond health-care quality.

## Introduction

As we continue to understand the role that social determinants of health play in affecting population health and well-being outcomes, the need to build capacity for systematic improvement in communities where people ‘are born, grow, live, work and age’ has never been more urgent. Initiatives such as Robert Wood Johnson Foundation’s Culture of Health Action Framework [1] and CDC foundation’s *Thriving Together* initiative [2] have enumerated the complex, interrelated dimensions of community health and well-being—e.g. health-care access, affordable housing, transportation, and poverty reduction—that must be addressed simultaneously for communities to thrive. Public Health 3.0—the US Department of Health and Human Services definition of the modern era of public health practice that emphasizes cross-sectoral collaboration to address the social determinants of health [3]—recommended shifting the focus of community public health efforts from being owned and delivered by public health agencies to being led by diverse community-based coalitions focused on local priorities and contexts.

**Key Messages**

- Building community organizations’ capacity to use QI methods is a promising approach to improving community health and well-being.
- Many studies described multifaceted interventions targeting health in minority and low-income communities.
- Few studies discussed how well traditional QI methods worked in community settings or whether adaptations were necessary.
- Organizations outside the community typically held decision-making authority.
- Additional research is necessary on whether community organizations can use QI methods independently to tackle complex public health problems that extend beyond health-care quality.

These recommendations, although timely and relevant, provide little concrete guidance on ‘tools’ that communities can use to advance their capability to improve health and well-being. Quality improvement (QI) methods (e.g. Lean, Six Sigma, or the Model for Improvement), used extensively to improve quality of care in health-care facilities, are promising. Although researchers interrogate the extent to which these methods can be causally attributed to improving outcomes in health-care settings, [4], there is little disagreement that QI methods’ emphasis on data-driven decision-making, local experimentation, and context-specific solution generation can strengthen health-care delivery processes if well-implemented [5]. Building the capacity of community organizations to use these methods could be a viable approach to developing local innovations that could tackle social determinants of health. For example, QI methods could guide community organizations to identify the multifaceted drivers of problems, develop localized solutions to address those drivers, test solutions rapidly on a small-scale, track data, and use those data to make informed decisions for improvement. However, the extent to which QI methods have been used to drive community-level improvements or whether community organizations engaged in improving health and well-being have knowledge and skill in these methods is unknown. This review aims to explore these questions, specifically:

1. How has community health improvement been defined?
2. What QI approaches have been used for community health improvement?
3. How are these approaches similar or different from those implemented in clinical settings (health-care improvement)?

## Methods

We used Batalden & Davidoff’s definition of QI: a ‘systematic approach to improve outcomes and systems by building the capability of communities to identify, prioritise and develop solutions to local systems problems’ [6]. Table 1 lists operational definitions of other key terms [7]. We used Arksey and O’Malley’s scoping review framework [8] with Levac, Colquhoun, & O’Brien’s proposed enhancements to conduct this review [9]. Our review protocol, in *BMJ Open*, is available at https://bmjopen.bmj.com/content/9/12/e034302. Because these review method details are published, we present an abridged account here.

**Table 1:** Operational definitions

Community	A group of people with diverse characteristics who are linked by social ties, share common perspectives, and engage in joint action in geographical locations or settings [12]
Community capacity	Knowledge, motivation, or skills to apply QI approaches to community well-being
Community setting	Where people eat, live, play, pray, or participate in other voluntary activities, where attendance/participation is not mandatory. For example, school site (or any site of mandatory activity) if outside of mandatory attendance hours; outcome is measured at school-level, but activities take place in community
Community well-being	Any health (physical, mental), educational, or social outcome measured at an aggregate level
Facility	School, correctional (juvenile, jail, prison), hospital, clinic, and military
Intervention	An activity, evidence-based program or policy that took place (i.e. is not merely proposed)
QI approach	Any QI method, such as Lean, PDSA, Six Sigma, or the Model for Improvement, or description of systematic process to improve community well-being

Our research team was comprised of a faculty member and three students (two doctoral and one undergraduate) in the School of Public Health with years of experience in QI practice and community health improvement.

### Inclusion and exclusion criteria

We reviewed peer-reviewed articles published in English from the USA, Australia, New Zealand, and Canada. We limited our review to these countries because of their similar national contexts. They are high-income countries that are part of the Anglosphere, with liberal market economies (in contrast to continental Europe’s more coordinated market economies), and that experience health disparities between their White/Caucasian racial majority and their minority including indigenous populations [10]. We considered studies published between 2000 and December 2019 because the use of systematic QI methods to improve health was limited prior to 2000, as the Institute of Medicine published the ‘Crossing the Quality Chasm’ report in 2001 that defined the six pillars of high-quality health care [11]. We placed no restrictions on study type. To be included, studies had to use QI approaches to address community-level well-being outcomes or a community’s capacity to improve in a community setting. Note that we did not place requirements on ‘who’ carried out the improvement work (e.g. community organization, community members, and institutions)—rather, this question was part of our findings. We excluded studies that (i) described interventions to improve quality but did not report using a systematic improvement method; (ii) did not focus on improving community health or well-being outcomes (e.g. study outcomes were improving program function, such as meeting attendance, without connection to a community well-being outcome, such as food security); and (iii) described QI efforts or interventions undertaken within a facility (e.g. a clinic) rather than in the community. Table 2 shows inclusion and exclusion criteria [7].

**Table 2:** Inclusion and exclusion criteria

Inclusion criteria: Population or problem: Well-being in community settings in the USA, Australia, New Zealand, or Canada. Intervention: Any intervention addressing improvement of well-being using a QI approach. Comparison: Any experimental or non-experimental study with or without comparison groups. Outcomes: Community-level well-being or community capacity to improve.
Exclusion criteria: Article focuses on drivers of improvement, effectiveness of improvement, etc., but does not use QI approach or describe QI processes. Article describes approaches to improve community, coalition, or program function (e.g. improve attendance of coalition members at meetings) without linkage to community well-being outcome. Intervention took place within the walls of a facility with no linkage to community setting.

### Data sources and search keywords

We identified relevant studies through Scopus, Web of Science, and Proquest Health Management databases. Our search strategy combined terms in three categories: (i) ‘community organizations’ (e.g. community coalitions or health departments); (ii) ‘QI methods’ enumerated by commonly used terms describing systematic QI approaches; and (iii) ‘health and well-being,’ described by terms including education, justice, and equity. Our protocol paper lists the complete search string details and justification for selecting data sources. We hand-searched references of studies we deemed relevant during full-text screening.

### Study selection

Our study selection involved three phases. In phase one, three authors (MWT, TC, and RR) reviewed 2% of titles and abstracts from extracted articles using the final search criteria. Using the inclusion criteria in Table 2, we designated studies as ‘eligible,’ ‘ineligible,’ or ‘maybe’ for full-text review. As we progressed through the 2% of title and abstracts, we discussed discrepancies in designations and adjusted interpretations of inclusion criteria. By completion of the review of the 2% of titles and abstracts, we reached an inter-rater reliability >80%. In phase two, one reviewer (RJ) reviewed the remaining titles and abstracts using the same inclusion criteria and designation strategy. Studies without abstracts were designated as ‘maybe’ if titles did not warrant immediate exclusion. In phase three, two authors (MWT and RJ) reviewed the full texts of each abstract designated as ‘eligible’ or ‘maybe,’ using the exclusion criteria to decide whether to exclude the study and documenting the reason. Through regular meetings with a third author (RR), we reached a consensus about studies where decisions on inclusion or reasons for exclusion differed.

### Data extraction and charting

We created the charting form after extracting data from the first few studies through consultations with the research team. We determined that identifying the role of the community and articulating the extent to which community members actively participated in study design or implementation were important. Therefore, one author (RR) created a customized data-extraction template that specified the institutional (i.e. university, government, or private organization) and community (i.e. community-based organization/individual, local health department/agency, or school) partners associated with the study and their roles.

We also reviewed the literature on collective impact [13] and Arnstein’s ladder of citizen participation [14] to develop meaningful categories to specify the locus of decision-making authority in each study. We created four categories: (i) institutional organizations (defined above), (ii) community organizations, (iii) multisectoral partnerships (multiple organizations and sometimes community residents collectively working toward an outcome), or (iv) community residents. Two authors (MWT and RJ) extracted the data; one author charted the data, and the second reviewed and amended the data with additional information or revisions in interpretation. Disagreements were resolved in regularly scheduled author meetings.

### Data synthesis and presentation

Data synthesis involved qualitative and quantitative components. We presented summary counts of included and excluded studies using the Preferred Reporting Items for Systematic Reviews and Meta-Analyses (PRISMA) flowchart and graphically summarized study characteristics (e.g. health area focus and QI method used). We recorded and tabulated each study’s community role and locus of decision-making authority. We presented data syntheses in tabular form.

In addition, three authors (MWT, RJ, and RR) independently synthesized the findings across studies to answer the research questions. We followed Braun, Clarke, Hayfield, & Terry [15] guidelines for thematic analysis (TA), that researchers should ‘use the approach to TA that is most appropriate for their research,’ and ‘use it in a “knowing” way’ to ‘produce an overall coherent piece of work’ (p. 7). First, each author individually listed salient themes from an integrated review of studies. Then, through consultation, we synthesized individual themes to identify overall findings and identified what is missing in published literature to set future research priorities.

## Results

**Figure 1:**
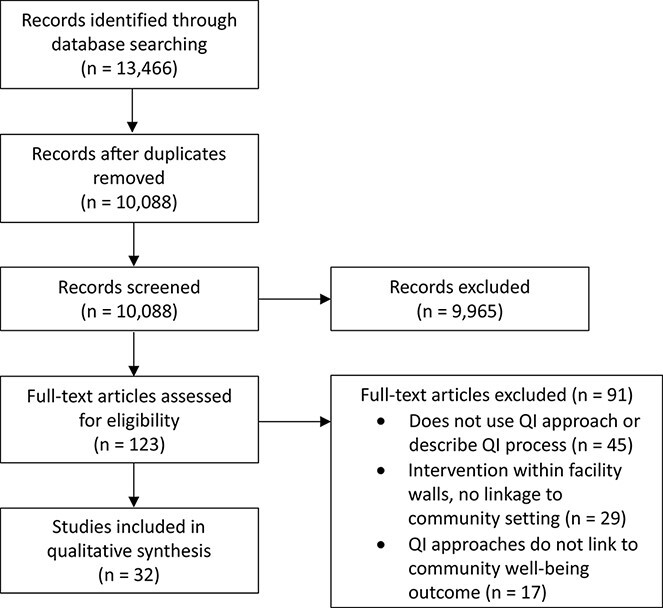
PRISMA flowchart of study selection.

**Figure 2:**
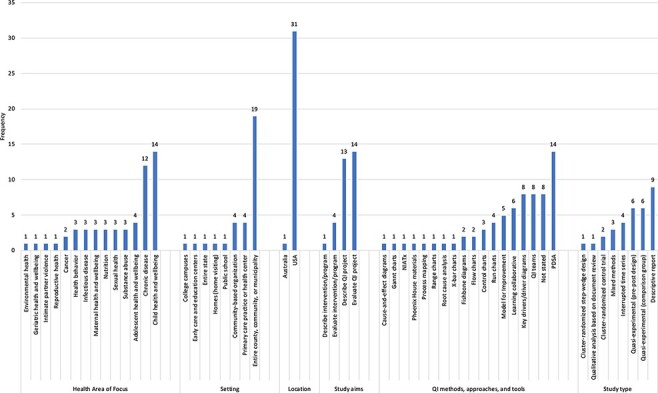
Summary of characteristics of studies in scoping review, the USA and Australia, 2004–2020.

Of the 10 088 unique articles identified through our database search, we deemed 9965 irrelevant during abstract/title screening (Figure 1). We initially selected 123 for full-text review. Within this set, we excluded 91 (45 for not using a systematic QI approach, 29 for not taking place in a community setting, and 17 for not targeting community well-being outcomes). We ultimately selected 32 studies for data extraction, listed in Table 3. Salient characteristics are summarized in Figure 2. 

### Characteristics of studies

#### Geography, settings, and focus areas

Figure 2 shows that nearly all the studies were set in the USA, encompassing 19 states and a wide geographic distribution. Interventions were implemented in a wide variety of community settings including Boys and Girls Clubs, YMCAs, home visits, indigenous communities, and low-income neighborhoods. The target groups for a significant majority of the studies were low-income and minority populations and emphasized mothers, youth, and adolescents. One study focused on the elderly, and one on indigenous communities.

Study focus areas split between those seeking to improve community health through prevention or promotion activities (19 studies) versus through chronic disease management (13 studies). Both groups included a diverse set of health topics and target populations across the lifespan. Examples of prevention projects included adolescent sexual health, substance abuse prevention, food insecurity, smoking cessation, adolescent mental health, immunization, breastfeeding, well baby care, healthy aging, and intimate partner violence. Chronic disease management topic areas were childhood obesity, substance abuse, diabetes, and pediatric asthma.

#### Interventions and use of QI

**Table 3:** Summary of articles included in scoping review, the USA and Australia, 2004–2020

Article	Intervention	Target of intervention	Outcome measures	Results	Community involvement	Institutional partners	Community partners	Locus of decision-making authority			
								Institutional org.	Community org.	Multisectoral partnership	Community residents
Chinman *et al*. [16]	Making proud choices: evidence-based program with learning modules on safe sex practices	Minority (primarily African American) adolescents living in Georgia and Alabama, USA	Sexual health knowledge, behaviors, attitudes	Improvements in condom measures; no statistically significant differences in sexual behavior outcomes	Getting to Outcomes staff train Boys & Girls Club staff to use QI for initiative targeting community	Getting to Outcomes: provided onsite training and implementation support	Boys & Girls Club: implemented intervention	X			
Mansour *et al*. [17]	SBHCs: offer comprehensive healthcare	Low-income and minority (African American) children attending public school institutions in Ohio, USA	Number of emergency department (ED) visits; percent of children with activity restrictions due to asthma	Statistically significant decreases in ED visits and asthma-induced activity restrictions	Schools and parents participated in a collaborative QI effort.	Philanthropic foundation: funded Hospital: helped implement initiative Institute for Health-care Improvement (IHI): served as a steering committee; provided program management, training, etc. FQHC: operated SBHCs	SBHC: staff and teachers contributed to workgroup City health department: provided staff, collaborated with workgroup			X	
Wiecha *et al*. [18]	A+: a QI toolkit created for YMCA afterschool programs to improve health promotion capacity	Youth living in New Hampshire, USA	Number of implementation sites meeting program standards	Scores and qualitative interviews demonstrate program-wide improvement and progress	A+ staff train YMCA staff to use QI for initiative targeting community	University: provided study review and approval A+ project staff: trained YMCA staff	Southern District YMCA: implemented intervention	X			
Dearinger *et al*. [19]	Diabetes self-management education: system-level intervention to improve glycemic control in adults	Rural and low-income communities in Kentucky, USA	Attendance per class, referral measures, availability, class content, etc.	Increased program outreach, program participation, enrollment, and referrals	University-trained local health departments to use QI in their community	University research group: supported training and QI team implementation at program sites State health department and health control program: administered program		X			
Stanhope *et al*. [20]	HealthMPowers: 3-year early care and education program that uses continuous improvement	Low-income and minority (African American) families and children in Georgia, USA	Capacity to improve, implementation of improvement plans and processes, child health outcomes	Reduced sugar-sweetened beverages in centers, improved food incentive offerings, increased taste testing	HealthMPowers staff trained early care and education centers to use QI for initiatives targeting families and children	HealthMPowers staff: provided training, technical assistance, resources, and evaluation for participating centers, and family events	Early care and education centers: implemented intervention, contributed to developing improvement plans	X			
Ariza *et al*. [21]	Promoting health project: aims to improve practice-based care for overweight and obese children	Low-income and minority (Hispanic) youth in Illinois, USA	Program satisfaction, obesity levels	High participant satisfaction, improvements in body mass index (BMI) levels	Three practices led QI efforts that included community outreach (referral systems)	Hospital, professional association: led the initiative Clinical practices: coordinated with local programs on intervention activities and programs	Community exercise and nutrition programs: engaged with institutional partners to implement program		X		
Brimblecombe *et al*. [22]	Good Food Systems Good Food for All Project: local, multisectoral group engages with existing governance structures when possible to improve food systems	Communities comprising Aboriginal and Torres Strait Islanders and non-Indigenous individuals, Australia	Community diet (sales of fruits, vegetables, confectionary items, soft drinks)	Declines in sales of confectionary items, slight increase in water; no clear trends in fruit, vegetable, or soft drink sales	Research team facilitated meetings where community stakeholders developed and co-monitored QI efforts	University: designed and led the project, and facilitated interactions with community groups	Community coordinator: liaised between community and research team Good Food Groups: helped facilitate intervention Community store: worked with intervention team, provided sales data				X
Cochran *et al*. [23]	Nevada Women’s Health Connection (WHC): a breast and cervical cancer screening program	Middle-aged and low-income women in Nevada, USA	Breast and cervical cancer screening enrollment	Increased enrollment and screening rates	University consultants taught health center staff QI, which they used for efforts including community outreach	University: helped design QI project State Department of Human Resources: implemented project Local American Cancer Society chapter: contracted case managers	Local clinics and providers: partnered in participant recruitment, enrollment, and screening County Health Department: contracted case managers	X			
Wright *et al*. [24]	Breast-feeding promotion in the Beaufort County Health Department’s WIC supplemental nutrition program	Mothers receiving WIC in North Carolina, USA	Number of women who begin or maintain breastfeeding	Increased number of mothers who engaged in breastfeeding	CPHQ taught the local health department QI, which it used for community outreach	Center for Public Health Quality (CPHQ): led a training program in health departments to improve ability and capacity to use QI	Health Department: staff served on QI team	X			
Felipe *et al*. [25]	ASTHO/CDC Heart Disease and Stroke Prevention Learning Collaborative: supports health systems and organizations to improve hypertension, focusing on systems change drivers	Communities in New York, Arkansas, and Oklahoma, (emphasis on indigenous communities in Oklahoma), USA	Hypertension prevalence (diagnosed and undiagnosed) and control rates	Improved hypertension control rates in NY; AK and OK established hypertension control and management programs (no outcomes reported in these states)	Members of a learning collaborative led QI efforts, which included community outreach	Federal agency: spearheaded initiative	State and tribal jurisdictions, CBOs: served as partners, developed QI plans Local health departments, public health agencies, and providers: served as partner institutions			X	
Beck *et al*. [26]	All Children Thrive Learning Network (ACT): network aiming to connect teams across sectors to encourage co-created solutions for child health equity	Children living in low-income neighborhoods in Ohio, USA	Hospitalization measures (e.g. inpatient bed-day rate, ED visit rate)	Decrease in inpatient bed-day rates in intervention groups; no decrease was observed in the control group	A hospital led a multidisciplinary team including community stakeholders in implementing QI.	Hospital: developed and led the intervention Health Network: assisted with team navigation and connection	Community-based individuals (e.g. families, social workers, providers, legal aids): helped carry out QI work, supported community-hospital relationships		X		
Fu *et al*. [27]	CDC taskforce 13 recommendations to improve immunization rates. Chronic Care Model: elements include community resources and policies, delivery system design	Low-income and minority (African American) children living in Washington, DC, USA	Coverage and timeliness of immunizations	Increased immunization rates and timeliness	Health centers implemented QI efforts that included community outreach		Health centers: sites of QI initiatives, provided staff for programs Local health departments: assisted in intervention, helped plan strategies		X		
Ford II *et al*. [28]	Bringing Healthy Aging to Scale: two evidence-based health promotion workshops on fall prevention and chronic disease self-management	Elderly populations in Wisconsin, USA	Number of workshops, workshop features (e.g. participants and number of enrollees)	Increased number of workshops, decreased risk behaviors, and emergency visits for fall-related injuries	County change teams used QI to increase workshops for community members	State Department of Health Services: provided staff for technical support	Not-for-profit program: administered the intervention; provided staff for technical support		X		
Kercsmar *et al*. [29]	Asthma Improvement Collaborative (AIC): aimed to improve pediatric patient health using the chronic care model framework	Low-income children and adolescents living in Ohio, USA	Asthma-caused ED visits and hospitalizations among target patient population	Decreased asthma-caused hospitalizations, ED visits, re-hospitalizations; increased percentage of population with ‘well-controlled asthma’	Hospital created improvement collaborative to implement QI, including community outreach.	Hospital: served as study site and assisted in project coordination and QI team organization; hospital institutional review board (IRB) team reviewed the study City health department: worked with hospital to implement intervention	Schools: worked with hospital and health department to implement intervention		X		
Kahn *et al*. [30]	Learning network: brings together the collective talents, ideas, and motivation of stakeholders across sectors to accelerate improvement	Youth and adolescents living in Ohio, USA	Infant mortality, hospital bed days, health perceptions, and reading proficiency	None reported	A learning network including community members carried out QI	Hospital: organized and coordinated the project	Network (consisted of CBOs, families, schools, etc.): executed QI initiatives and projects			X	
Gerding *et al*. [31]	Environmental Public Health Performance Standards (EnvPHPS) Version 2.0: standards describing activities an environmental public health program should conduct	Entire communities across 14 states in the USA	Implementation and effectiveness of vector-control programs	Control programs implemented throughout health departments varied in topics and goals; projects addressed a number of policy goals	local health departments carried out QI initiatives including community outreach	Federal agency: funded Not-for-profit: provided funding; helped identify areas for improvement and QI projects Research institute: identified health departments for the intervention	CBOs (businesses, radio stations, tribal/ local agencies, dealerships, etc.): helped with intervention/ QI Local health departments: delivered intervention/QI		X		
Brown *et al*. [32]	Infant medical home: well-child visits during first 4 months of life. Evidence-based home visiting	Low-income infants and children in the USA	Patient age when attending visits, timeliness of visits	Decline in the mean newborn visit age in all clinics; mixed results for timeliness	Researchers chose clinics to form QI improvement teams. Teams consulted with community collaborators in choosing solutions	Home-visiting agency: collaborated with clinic to enroll participants in the program Hospital: provided research capabilities such as funding and data analysis	Community collaborators (e.g. education centers, community service groups): engaged in QI, helped identify solutions Clinics: helped design intervention	X			
Indyk and Indyk [33]	Database management and reporting system: used to collect, analyze, and report data for evaluation, QI, and external reporting requirements	Low-income individuals and families living with HIV in New York, USA	Medical service access and health-care outcomes	None reported	Organizations use a database system to track community outreach and use the data for improvement		Health and social services agency, outpatient clinic, hospital-based clinic: implemented interventions and QI; collected data; maintained databases		X		
Bharel *et al*. [34]	Cervical cancer screening	Low-income and homeless women living in Massachusetts, USA	Cervical cancer screening rates	Increased cervical cancer screening rates	Not-for-profit led improvement efforts including community outreach		CBOs (health fairs, homeless shelters): helped deliver intervention Not-for-profit: helped develop intervention		X		
Lanter *et al*. [35]	A variety of interventions based on strategic needs and resources available at each school	College students attending universities throughout the USA	Harm measures, encounter rates with medical services and law enforcement	Increased number of interventions aimed at reducing high-risk drinking across college campuses	College campus community stakeholders participated in a learning collaborative to implement QI efforts	Universities: carried out intervention activities Learning Collaborative on High-Risk Drinking (LC-HRD): provided support and training to colleges and universities in QI methods	Community stakeholders: participated in learning collaborative, QI implementation				X
Woodhouse *et al*. [36]	Childhood Asthma Management Program: 5 organizations proposed various strategies to control asthma	Low-income rural and urban communities in Georgia, USA	ED visits, missed school days, school nurse visits	None reported	University provided community organization grantees technical assistance and evaluation support for QI	Foundation: initiated intervention University: provided research services, evaluation, technical support, and assistance with QI activities	Community organizations (school system, community home visitation program, etc.): carried out intervention	X			
Spratt *et al*. [37]	Durham Diabetes Coalition: created a geographic health information system to address individual and community health	Low-income minorities (African Americans) living in North Carolina, USA	Health-care outcomes (e.g. hospitalizations, ED visits, mortality)	None reported	Community organization created a geographic health information system, used for community-targeted QI		Community organizations and agencies, health system, county health department, FQHC, etc.: participated in coalition		X		
Fisher *et al*. [38]	Chronic care model: frame of reference for multicomponent systems to support productive patient–provider interactions	Low-income and minority (African American) communities in St. Louis, USA	Availability of smoking cessation resources in communities and neighborhoods	Higher levels of neighborhood resources and support	Clinic implemented QI efforts that included community outreach		FQHCs: implemented intervention and QI		X		
Grossman *et al*. [39]	Chronic Care Model (CCM): a guide to QI and disease management activities for chronic medical conditions	Entire communities	Number and types of interventions undertaken by health centers	Few activities were fully implemented and evaluated; low number of activities with high impact; interventions frequently targeted developing community linkages	IHI-led community health centers in a learning collaborative to teach QI, which centers used for initiatives including community outreach	Government agency: sponsored intervention and provided technical assistance Institute for Healthcare Improvement (IHI): provided training and technical support; helped lead the intervention Research group: provided data analysis and research services	Community health centers: implemented the intervention and QI program	X			
Inkelas *et al*. [40]	Magnolia Community Initiative: multiple sectors and programs build a system of care for families that can change outcomes in a geographic population	Low-income children and families living in California, USA	Measures of childhood well-being	None reported	Network of community and government organizations implemented a collective QI effort targeting population outcomes	Government partners (county officials, health departments, etc.): members of network carrying out improvement work	CBOs (family support programs, educational programs, etc.): members of network carrying out improvement work			X	
Allegheny County, Maternal and Child Health Care Leadership Collaborative, Keyser, & Pincus [41]	Learning collaborative: aimed to develop a model system of care for mothers and young children in the region	Low-income mothers and children living in Pennsylvania, USA	Program enrollment, screening, assessment, and referral rates	Increased screening and treatment referrals	County health department convened a multisectoral learning collaborative that implemented QI efforts targeting the community	University-Think Tank Collaborative: provided support and research services for the learning collaborative	County health department: organized collaborative Lawmakers, providers, community-members, etc.: participated in collaborative			X	
Riley *et al*. [42]	QI collaborative: Health departments were selected to create a cross-departmental local team; each department’s director was encouraged to participate in each project	Varied	Nature, extent, and impact of QI projects	QI projects were implemented for a number of core processes (e.g. sexually transmitted diseases, child health); nearly 40% of projects’ metrics improved >25%	Local health departments used QI for projects including community-targeted efforts		Collaborative (composed of state and local health departments): collaborated to implement and carry out QI activities		X		
Grow *et al*. [43]	ACT! Actively Changing Together: hospital-community organization partnership	Families living in Washington State, USA	Changes in health behaviors and attitudes, health and well-being outcomes (e.g. fitness, home environment changes, quality of life)	Statistically significant improvement for metrics such as patient-reported home environments, quality of life, satisfaction, and BMI	Community organization and hospital used QI to improve program targeting community	Hospital: designed and implemented program Federal agency: provided funding	YMCA of Greater Seattle: worked with hospital to develop and implement the program		X		
Chinman *et al*. [44]	Council of Alcoholism and Drug Abuse: operates 16 adult and adolescent substance abuse prevention and treatment programs	Staff and clients of a drug abuse program in California, USA	Examples include nature of QI actions, progress within PDSA cycle, resources and collaborations required for QI actions	QI interventions mostly targeted program staff; only 2 targeted clients (e.g. recruitment). 63% of programs completed PDSA cycles	Research team taught community organization QI, which staff used for initiatives including community outreach	Research team: provided technical support, resources, training, etc. and assisted in QI workshop implementation	Council network: organization composed of several substance abuse prevention and treatment programs	X			
Crane *et al*. [45]	Maternal Opiate Medical Supports Project: offer person-centered behavioral health & obstetric care for pregnancy, childbirth, and postpartum	Low-income pregnant women living in Ohio, USA	Utilization and retention rates, birth and stability outcomes	Increased retention, counseling, treatment participation, and decreased out-of-home placement	Clinical experts trained local clinical organizations to use QI for an intervention that included community outreach	State officials (Governor’s office, health and human service agencies, State Dept. of Medicaid, State Dept. of Mental Health and Addiction): trained clinical organizations	Clinical organizations: carried out intervention/QI Community providers: partnered with clinical organizations	X			
Agu *et al*. [46]	National Maternal, Infant, and Early Childhood Home Visiting: provides home visiting for pregnant women and families with young children	Pregnant women with young children living in Florida, USA	Screening and referral rates	Increased screening and referral rates	Research team developed a change package that home visiting agencies implemented using QI	Researcher ‘faculty’ team: team composed of researchers in violence and injury prevention and home agency representatives	Home-visiting programs: implemented intervention	X			
Chinman, Ebener *et al*. [47]	CHOICE: 5-session evidence-based alcohol and drug prevention program. Getting to Outcomes: implementation support intervention	Adolescents living in California, USA	Substance use attitudes, intentions	No observed differences in attitudes/intentions between the two groups	Getting to Outcomes staff trained Boys & Girls Club staff to use QI for initiative targeting community	Getting to Outcomes: provided training, resources, and technical assistance to the Boys & Girls Club	Boys and Girls Club: implemented intervention	X			
								13	12	5	2

SBHC—School-Based Health Center; FQHC—Federally-Qualified Health Center; WIC—Women, Infants, and Children; CBO—Community-Based Organization.

Many studies used QI methods to generate solutions (frequently community outreach) to improve implementation of

Box 1.

- Diabetes education: Diabetes self-management education (DSME): a public health, system-level intervention to improve glycemic control in adults. In six local health departments, facilitators trained a QI team and helped them develop and implement a 9- to 12-month QI project in their community to improve DSME services [19].
- Early childhood care: HealthMPowers is a 3-year early care and education (ECE) program that uses continuous improvement to provide training, improve programs, measure impacts, and sustain partnerships. Sixty-five ECE centers in Georgia formed a team that implemented annual self-assessments and improvement plans, such as improving home environments [20].
- Pediatric asthma: The Asthma Improvement Collaborative enhanced pediatric asthma care, e.g., by strengthening community and hospital relationships. A multidisciplinary improvement team developed a key driver diagram of emergency department use by the target population and tracked outcomes using control charts [29].

programs, guidelines, or standards. A few used QI to develop local interventions. While project team members received QI training in most studies, the training objective was to apply project-specific QI methods, rather than to build general QI expertise that could apply to other community improvement efforts. Box 1 shows typical QI use examples.

#### Institutional and community roles

Nearly all selected studies relied on external institutional partners (e.g. university, technical service provider, or federal or state agency) for funding, planning, training, supervision, and/or evaluation. Community organizations (e.g. YMCAs, schools, and local health departments) were involved in 31 of the 32 studies but did not always have decision-making authority and often were involved only in implementing interventions. Moreover, since community organizations typically were local chapters of state or national institutions (e.g. YMCA), the extent to which the local chapters truly were integrated into and reflect the local community was not always clear. Table 3 describes the distribution of decision-making authority across studies. Institutional partners had decision-making authority over priorities and interventions in 13 studies, community organizations in 12, and multiple stakeholder organizations (which could include community organizations and community residents) in 5. Only 2 studies were designed to ‘center’ decision-making authority about interventions directly within the community.

#### QI methods and research designs

QI research study designs varied in rigor and in the types of designs used. Three studies used randomized designs. Most used quasi-experimental designs of varying strength: six used comparison groups, five used interrupted time series, and six used pre–post designs. Ten studies used narrative descriptions of projects. Two employed mixed methods. Overall, detailed information about how QI study activities were implemented was lacking.

#### Outcomes

As Table 3 shows, 18 studies used project-relevant outcome measures (e.g. related to sexual behavior, emergency department visits, hypertension control, cervical cancer screening, and breastfeeding behavior). Some of these studies also used process variables proximal to the measured outcomes, such as availability of sugar-sweetened beverages, attendance at diabetes self-management classes, or satisfaction with obesity prevention programs. Fourteen studies exclusively used process measures, including implementation variables such as the number of workshops conducted or the number of sites conforming to performance standards. Five studies did not report any results. Twenty-five of 27 reported positive change at the end of the QI interventions; two studies reported null results between intervention and comparison groups. Because statistical analysis of outcomes was sparsely reported, it was not possible to assess whether positive results that were reported were significant, could be attributed to the intervention, or reflected selective reporting by the authors.

## Discussion

### Principal findings

We report our principal findings by the research questions described earlier.

1. ‘How has community health improvement been defined?’ All the studies defined health improvement in terms of management of chronic diseases or health promotion activities. This focus is substantively different from improving the quality and safety of patient care, which has been the primary emphasis of QI to date in the health sector. Moreover, the studies described complex, multifaceted interventions that involved education, behavior change, and modifications to service delivery processes. This has not historically been the focus of QI initiatives in clinical settings, which are more narrowly focused on clinical interventions. The current growth and interest in Learning Healthcare Systems [48] and in Learning Health Networks [49] that enable collaborations between patients, families, and care teams to address the entire system of care for a patient have begun to shift this paradigm in the health-care space, but the emphasis is still on providing care after patients have been diagnosed. The health promotion or public health aspects of some of the included studies differentiate the notion of ‘improvement’ in community settings.
2. ‘What QI approaches have been used for community health improvement?’ The Model for Improvement (MFI) [50] was the most common improvement method, mentioned in five studies. Fourteen studies mentioned the use of Plan-Do-Study-Act (PDSA), although some of these may have used PDSA and MFI as synonyms. Breakthrough collaboratives or other learning networks were used in six studies. Individual tools such as driver diagrams [51], flowcharts, run charts [52], and cause-and-effect diagrams also were mentioned, as shown in Figure 2. Scant detail was provided on how exactly the QI methods were used. Eight studies left specific QI methods, approaches, or tools unstated.
3. ‘How are these approaches similar or different from those that have been implemented in the clinical setting (health-care improvement)?’ No new methods were developed specifically for community health improvement. Several studies applied health-care QI methods to complex, multicomponent interventions. However, there was little discussion on how well these worked in community settings or how to adapt health-care methods for typically encountered community setting situations (e.g. no routinely collected electronic medical record data; no clearly defined protocols for interventions; QI teams that are coalitions and not employees of clearly defined health systems). Overall, comparison between community and health-care QI methods was challenging because of the lack of detail about how QI activities were implemented in the included studies, which is a common problem in QI studies [53].

### Strengths and limitations

To our knowledge, this review is the first to study the use of QI methods in community settings. However, because these settings are not clearly defined, we needed to create operational definitions for what constituted community improvement, and the studies we selected were based on these definitions. Other definitions for community health improvement may result in other studies being included. Moreover, our study only included peer-reviewed literature. It is possible that community organizations are engaged in QI projects that have been documented in websites, donor reports, or conference presentations that have not reached academic journals. Conducting a similar review including the gray literature would likely produce a larger body of work than we have identified in this review.

### Interpretation within the context of the wider peer-reviewed literature

Since QI for health-care improvement is a mature field, we expected to identify a body of literature demonstrating how QI researchers have adapted these methods for use in more complex, distributed, and data-poor community settings. Our selected studies failed to address the complex nature of community health in two critical ways. First, while our search criteria intentionally included articles addressing both health and well-being, most of the studies emphasized only physical aspects of health. They were conceptualized as extensions of hospital-based QI efforts that focus on improving clinical outcomes or enhancing operational care delivery processes. The World Health Organization recognizes that community well- being extends beyond physical health and includes mental and social aspects [54]—all of which should be the scope of community health improvement.

Second, while most of the studies focused on low-income and socially disadvantaged populations, few addressed the social determinants of community health or explicitly acknowledged structural factors that affect outcomes. These factors include income inequality, mass incarceration, and structural racism [56]. Papers that did focus on structural factors were Brimblecombe *et al*. [22], which addressed system drivers of food insecurity, and Inkelas, Bowie, and Guirguis [40], which described a network of organizations using QI to improve population outcomes such as child well-being through multisectoral collaboration.

### Implications for practice, policy, and research

Our findings indicate the need for more research on the applicability of QI methods on the social determinants of health and well-being in allied systems such as education and housing. We must build knowledge about how to define and measure outcomes, collect process data, and test and implement interventions to tackle these complex problems.

We also must learn how to engage community residents with deep local knowledge as an integral part of community improvement efforts; the predominantly top-down approaches we found in this review may impede improvement in underserved and marginalized communities. QI teams in community settings must be assembled, organized, and managed differently from clinical teams. There is little peer-reviewed, academic literature about how this should be done.

Involving community members should include much more than just assembling teams. Community-led QI initiatives should be based on principles of Collaborating for Equity and Justice [57], with the goal of building resident leadership to enable community members to set an improvement agenda focused on systems’ change, not just unitary outcomes. These principles are echoed in other community-led, equity-based approaches such as community-based participatory research and design justice, with the tenet of ‘nothing about us without us’ [58]. Embedding QI capabilities into communities should be an intentional focus of community health improvement efforts and is an area of research that is not reflected in the peer-reviewed literature.

### Implications for future documentation

Finally, this study shone light on a potential gap between improvement work that may be undertaken by communities and what is published in peer-reviewed literature. As we have indicated, the 32 peer-reviewed papers that met our inclusion criteria document studies that have been led by researchers and academic implementers because these are the ones with the resources and incentives to engage in formal documentation efforts and the peer review. Community-led improvement initiatives that may have been documented locally as project reports or as presentations for stakeholders would not have made it into the peer-reviewed literature that we reviewed and could represent a bias in our findings. To expand the documentation of community-based efforts, accessible methods need to be developed for communities to synthesize and report on findings and learning. A recent example of such an effort is the participatory synthesis process that was used in the Robert Wood Johnson Foundation–funded *100 Million Healthier Lives* initiative in which community implementers partnered with evaluation team to document generalizable insights from routine program data [55, 59]. The process of synthesis, documentation, review, and publication in peer-reviewed journals was arduous and time-consuming and required a commitment well beyond the funds provided by the grant. To accelerate and facilitate the process of dissemination from the field, journals need to create accessible and inexpensive options for dissemination. While a few journals have begun to publish field reports (e.g. *BMJ*’s Quality Improvement Reports), the submission process has an academic focus that many community practitioners may find burdensome and not worth the effort.

## Conclusion

Public health has recognized the need to go beyond its traditional boundaries and to engage cross-sectoral collaborations to address social determinants of health. Our scoping review indicates that few published community health improvement initiatives extend beyond single-population health outcomes to address multifaceted systems’ change. Details are scarce about how to adapt existing QI methods to these contexts or whether new methods should be created. More importantly, decisions to use QI methods for community health are not yet in the hands of community members. As the coronavirus disease 2019 era has shown, common restrictions imposed at the state or county level result in widely varying results at the community level [60]. While communities are subject to the same constraints, their infection processes are widely different and therefore require different, context-appropriate containment solutions. Communities urgently need to be actively involved in developing solutions to improve health and well-being. Our scoping review shows that community health improvement that has been published in peer-reviewed literature is still primarily focused on providing clinical care in community settings, with some progress in implementing interventions that reach whole populations—a finding that may reflect bias in what gets published rather than work happening on the ground. There is much work to be done.

## Data Availability

The data underlying this article are available in the article and in its online supplementary material.
